# Detection of skin α-synuclein using RT-QuIC as a diagnostic biomarker for Parkinson’s disease in the Chinese population

**DOI:** 10.1186/s40001-024-01705-x

**Published:** 2024-02-09

**Authors:** Jiaqi Li, Suying Duan, Jing Yang, Honglin Zheng, Yanpeng Yuan, Mibo Tang, Yanlin Wang, Yutao Liu, Zongping Xia, Haiyang Luo, Yuming Xu

**Affiliations:** 1 Department of Neurology, The First Affiliated Hospital of Zhengzhou University, Zhengzhou University, Zhengzhou, Henan China; 2 The Academy of Medical Sciences of Zhengzhou University, Zhengzhou University, Jian-She East Road, Zhengzhou, 450000 Henan China; 3 Department of Geriatrics, The First Affiliated Hospital of Zhengzhou University, Zhengzhou University, Zhengzhou, Henan China; 4 Henan Key Laboratory of Cerebrovascular Diseases, The First Affiliated Hospital of Zhengzhou University, Zhengzhou University, Zhengzhou, Henan China; 5 Institute of Neuroscience, Zhengzhou University, Zhengzhou, Henan China

**Keywords:** α-synuclein, RT-QuIC, Skin, Parkinson’s disease

## Abstract

**Background:**

Several studies have indicated that skin holds promise as a potential sample for detecting pathological α-Syn and serving as a diagnostic biomarker for α-synucleinopathies. Despite reports in Chinese PD patients, comprehensive research on skin α-Syn detection using RT-QuIC is lacking.

**Objective:**

This study aimed to evaluate the diagnostic performance of skin samples using RT-QuIC from PD patients in the Chinese population.

**Methods:**

Patients with sporadic PD and controls were included according to the British PD Association Brain Bank diagnostic criteria. The seeding activity of misfolded α-Syn in these skin samples was detected using the RT-QuIC assay after protein extraction. Biochemical and morphological analyses of RT-QuIC products were conducted by atomic force microscopy, transmission electron microscopy, Congo red staining, and dot blot analysis.

**Result:**

30 patients clinically diagnosed with PD and 28 controls with non-α-synucleinopathies were included in this study. 28 of 30 PD patients demonstrated positive α-Syn seeding activity by RT-QuIC assay. In contrast, no α-Syn seeding activity was detected in the 28 control samples, with an overall sensitivity and specificity of 93.3% and 100%, respectively (*P* < 0.001). Biochemical characterization of the RT-QuIC product indicated fibrillary α-Syn species in PD-seeded reactions, while control samples failed in the conversion of recombinant α-Syn substrate.

**Conclusion:**

This study applied RT-QuIC technology to identify misfolded α-Syn seeding activity in skin samples from Chinese PD patients, demonstrating high specificity and sensitivity. Skin α-Syn RT-QuIC is expected to be a reliable approach for the diagnosis of PD.

**Supplementary Information:**

The online version contains supplementary material available at 10.1186/s40001-024-01705-x.

## Introduction

Parkinson’s disease (PD) is a neurodegenerative disorder characterized by the presence of α-Synuclein (α-Syn) inclusions known as Lewy bodies (LBs) [1]. The definitive diagnosis of PD currently relies on the post-mortem detection of α-Syn-containing LBs in the brain, making antemortem diagnosis unfeasible. To overcome this limitation, there is an urgent need to develop methods for detecting α-Syn in peripheral tissues. Real-time quaking-induced conversion (RT-QuIC) assays have emerged as ultrasensitive protein amplification assays for detecting α-Syn, initially developed for prion disease detection [2, 3]. These assays take advantage of the prion-like properties of misfolded α-Syn to achieve highly sensitive detection in various tissues and biological fluids, including the brain, cerebrospinal fluid (CSF), submandibular gland, and skin [3–8]. However, the invasiveness of lumbar puncture, relative contraindications, and limited patient acceptance restrict its widespread use. Similarly, submandibular gland biopsies are associated with potentially severe side effects.

The skin, which derives embryonically from the same germ layer as the central nervous system (CNS), holds the potential as a minimally invasive approach for diagnosing PD. Phosphorylated α-Syn depositions in autonomic nerve terminals from the skin of PD patients were detected through immunohistochemistry and immunofluorescence [9]. Skin biopsy can be easily repeated and performed at all stages of the disease, making it an excellent candidate sampling for PD diagnosis. Recent studies have explored the application of skin RT-QuIC in detecting misfolded α-Syn in PD and have reported high sensitivity and specificity in identifying pathological α-Syn aggregates associated with PD [6, 8, 10]. Such results have generated significant interest and enthusiasm in the scientific community for further exploring RT-QuIC’s diagnostic utility. In the present study, we successfully applied RT-QuIC technology to analyze skin samples from a cohort of Chinese PD patients.

## Methods

### Participants

All subjects gave written informed consent and the study was approved by the hospital medical ethics committee with ethics number: 2022-KY-0386-002. Finally, the study confirmed to the declaration of Helsinki.

Patients with sporadic PD and controls with non-α-synucleinopathies were enrolled based on the diagnostic criteria set forth by the British PD Association Brain Bank. PD inclusion criteria: (1) meeting the diagnostic criteria of the British PD Association brain bank; (2) exclusion of familial PD and Parkinson-Plus syndrome; (3) consent to sign the informed consent form and retain the peripheral skin. PD exclusion criteria: (1) not meeting the diagnostic criteria of the British PD Association brain bank; (2) not being able to exclude familial PD or Parkinson-Plus syndrome; (3) refusal to sign the informed consent form or retain the peripheral skin.

The inclusion and exclusion criteria for the control group were matched to the sex and age of the PD patient group. The controls were carefully examined and did not have parkinsonism or other neurological diagnosis (e.g., cognitive, motor, autonomic, or olfactory dysfunction). They included healthy individuals with physical examinations at the First Affiliated Hospital of Zhengzhou University and patient populations treated in the neurology department for unrelated diseases. These control participants had no previous history of PD, multiple system atrophy (MSA), dementia with Lewy bodies (DLB), pure autonomic failure (PAF), or REM sleep behavior disorder (RBD). Furthermore, they had no family history of neurological disorders, including head trauma, encephalitis, cerebrovascular disease, or Alzheimer’s disease (AD).

All PD patients were assessed for the severity of motor symptoms based on Hoehn and Yahr stage and the MDS-UPDRS III rating scale. Additionally, they all underwent cognitive assessment using the Mini-Mental State Examination (MMSE) scale.

#### Skin biopsy procedure and sample preparation

Skin samples (approximately 3 × 3 mm each in size) were collected from the posterior cervical region of patients with PD and controls. The samples were trimmed to 25 mg and stored at − 80 °C. Before processing, the samples were washed 3 times in Tris-buffered saline (TBS) containing 10 mM Tris–HCl and 133 mM NaCl (pH 7.4). The skin homogenates at 10% (weight/volume) were prepared in skin lysis buffer containing 2 mmol of calcium chloride and 0.25% (weight/volume) collagenase A (Roche) in TBS and incubated in a shaker at 37 °C for 4 h, followed by homogenization with a bullet blender (JXFSTPRP-24) with 0.5-mm zirconium oxide beads at 65 HZ frequency for 2 min. After homogenization, samples were centrifuged at 500 g for 5 min and the supernatant was used to make serial tenfold (w/v) dilutions.

#### Purification of recombinant α-Syn protein

Purification of recombinant α-Syn protein was performed as described previously with minor modifications [11]. In brief, human α-Syn-expressing cells were cultured in LB media. After protein induction with IPTG, the cells were harvested, and the protein was extracted using the high salt buffer and protease inhibitors. Unwanted proteins were precipitated and removed by boiling and centrifugation. The α-Syn protein was then concentrated, filtered, and loaded onto a Superdex 200 column. Later, fractions positive for recombinant α-Syn were combined, concentrated, and 0.2 μm filtered before loading onto a Hi-trap Q HP anion exchange column. Fractions containing α-Syn were collected, pooled, and dialyzed. After buffer exchange and filtration, the protein was lyophilized and stored at – 80 ℃.

#### RT-QuIC analysis

RT-QuIC analysis was conducted as described previously [12], with minor modifications. Briefly, the RT-QuIC reaction mix was composed of 40 mmol of phosphate buffer (pH 8.0), 170 mmol of sodium chloride, 0.1 mg/mL of recombinant human wild type α-Syn, 20 μmol of thioflavin T (ThT), and sodium dodecyl sulfate, 0.001%. Each reaction consisted of 2 μL of skin homogenate (0.245–0.5 μg/μL) as a seed and 98 μL of α-Syn RT-QuIC reaction mixture with six 0.8 mm silica beads (OPS Diagnostics, Lebanon, NJ) per well in a 96-well plate (Nalgene Nunc International, 265301). The plates were closed with a plate sealer film (Thermo Fisher) and incubated at 42 °C in a BMG FLUO star Omega plate reader. The incubation plates were subjected to cycles of 1 min shaking (400 rpm double orbital) and 1 min rest for at least 70 h. ThT fluorescence measurements (450 ± 10 nm excitation and 480 ± 10 nm emission; bottom read) were taken every 30 min. Each sample was tested in triplicate, and most of the positive samples were amplified in all three of their technical replicates. Threshold ThT fluorescence was calculated by statistical results. For a sample to meet the criteria for being considered positive, the fluorescent signal exceeded the threshold in at least 50% of the replicate wells (e.g., ≥ 2 of 3) before 70 h.

#### Transmission electron microscopy (TEM) and atomic force microscopy (AFM)

TEM: Removed a 10 μL sample and applied it to a copper grid (200-mesh, carbon-coated film from Zhongjing Scientific Instrument) for 1 min for sedimentation. Eliminated excess liquid using filter paper. Applied 10 μL of 2% uranyl acetate to the copper grid, let it sediment for 1 min, and removed excess liquid with filter paper. Air-dried at room temperature for several minutes. Performed transmission electron microscopy (TEM) imaging at 80–120 kV to obtain results.

AFM: Retrieved 50 μL of the sample stored in a − 20 °C freezer and transferred it to a centrifuge tube. Diluted it tenfold by adding Milli-Q water. Took 25 μL of the diluted solution and dropped it onto a clean mica sheet (Ted Pella Inc, USA). Allowed it to air-dry and then placed it on the AFM sample stage. Conducted testing using the intelligent mode “Scan Asyst in the air” with an AFM.

#### Congo red staining

Placed 2 μl of the productions on a glass slide and dried at room temperature. Stained with 0.5% (w/v) Congo Red solution for 20 min, followed by rapid washing with 100% absolute ethanol. Dehydrated the glass slide twice in 100% xylene (5 min each), and mounted with a neutral mounting medium. Captured images under both natural and polarized light.

#### Dot blot

The final a-Syn RT-QulC products from each well of the RT-QuIC assay plate were allowed to load onto a nitrocellulose membrane (0.2 µm pore) for 1 h. The nitrocellulose membrane was blocked with non-fat dry milk (5% in TBST) for 1 h at room temperature (RT). The membrane then was incubated with a rabbit monoclonal (MJFR-14-6-4-2) α-Syn filament conformation-specific antibody (dilution 1:1000) and alpha-synuclein rabbit polyclonal antibody (Proteintech, USA) for 1 h at RT, and triple-washed with 1X TBST for 10 min each. The membranes then were incubated with the secondary antibody (Amersham goat Anti-Rabbit IgG-HRP) made in non-fat dry milk (5% in TBST) (dilution 1:5000) for 30 min at RT followed by 3 washes with 1X TBST. Later, membranes were scanned with a LI-COR machine and densitometric quantification dots was done using ImageJ software.

### Statistical analysis

For statistical analysis, SPSS26.0 (SPSS Inc., IBM, USA) program was used. Raw data were analyzed using Student’s two-sample unpaired t-test. Asterisks were assigned as follows: *P ≤ 0.05, **P ≤ 0.01, ***P ≤ 0.001 and ****P ≤ 0.0001. The number of biological replicates is expressed as “n” unless otherwise mentioned. GraphPad 8.0 was used for multi-variate correlation analysis.

## Results

### Demographics

In this study, a total of 30 patients with PD and 28 controls were included. The specific clinical characteristics are shown in Table 1. The average age of the PD group was (63.3 ± 9.5) years, while the control group had an average age of (61.1 ± 11.5) years. The male-to-female ratio in the PD group was 16:14, whereas, in the control group, it was 12:16. The duration of the disease in the included PD patients was (5.4 ± 3.1) years.

**Table 1:** Demographic characteristics and skin RT-QuIC assay fluorescence of different groups

Variable	PD	Control
Age, mean (SD), y	63.3 (9.5)	61.1 (11.5)
Samples, n	30	28
Male sex, n (%)	16(53.3)	12(42.9)
Disease duration (from diagnosis), mean (SD), y	5.4(3.1)	
H&Y,^a^ mean (SD)	2.5(0.9)	
MDS-UPDRS III score,^a^ mean (SD)	43(16.6)	
MMSE,^a^ mean (SD)	21.5(7.2)	
RT-QuIC assay		
No. of samples analyzed, mean (SD), %	30	28
* P* (compared with control)	*P* < 0.001	

SD, standard deviation; H&Y, Hoehn and Yahr; MDS-UPDRS III, Movement Disorders Society–Unified Parkinson’s Disease Rating Scale Part III; MMSE, Mini-Mental State Examination

^a^Only patients who can determine disease duration and clinical scales of disease severity are counted

### Detection of α-Syn seeding activity in skin biopsy Samples from patients with PD

Before conducting RT-QuIC experiments, we systematically evaluated experimental conditions and identified a significant impact of sodium dodecyl sulfate (SDS) on the reaction’s sensitivity and specificity. SDS, acting as a surfactant, was tested at concentrations of 0%, 0.0005%, 0.00075%, 0.001%, and 0.00125%. The positive control used 2 μl of a 10^–3^ α-Syn fibril (artificial seeds), while the negative control employed PBS. Results (Additional file 1: Figure S1) showed a notable increase in ThT fluorescence in the positive control after 10 h. Furthermore, ThT fluorescence exhibited a positive correlation with SDS concentration in the positive control. Specifically, 0.00125% SDS induced an early fluorescence onset and the highest plateau. Only the negative control with 0.00125% SDS showed a slight increase in ThT fluorescence, while others remained at baseline in RT-QuIC reactions. The presence of 0.00125% SDS in the negative control resulted in a mild increase in ThT fluorescence, suggesting a potential for false positives. To ensure both sensitivity and specificity, 0.001% SDS was chosen as the concentration for the RT-QuIC reaction.

After establishing suitable experimental conditions, we applied the RT-QuIC assay to examine the aggregation seeding activity of α-Syn in skin samples. The samples were divided into two cohorts: Cohort 1, comprising 4 PD patients and 4 controls, was utilized for initial RT-QuIC testing. Cohort 2, consisting of 30 PD patients and 28 controls, was employed for subsequent research and analysis.

**Fig. 1:**
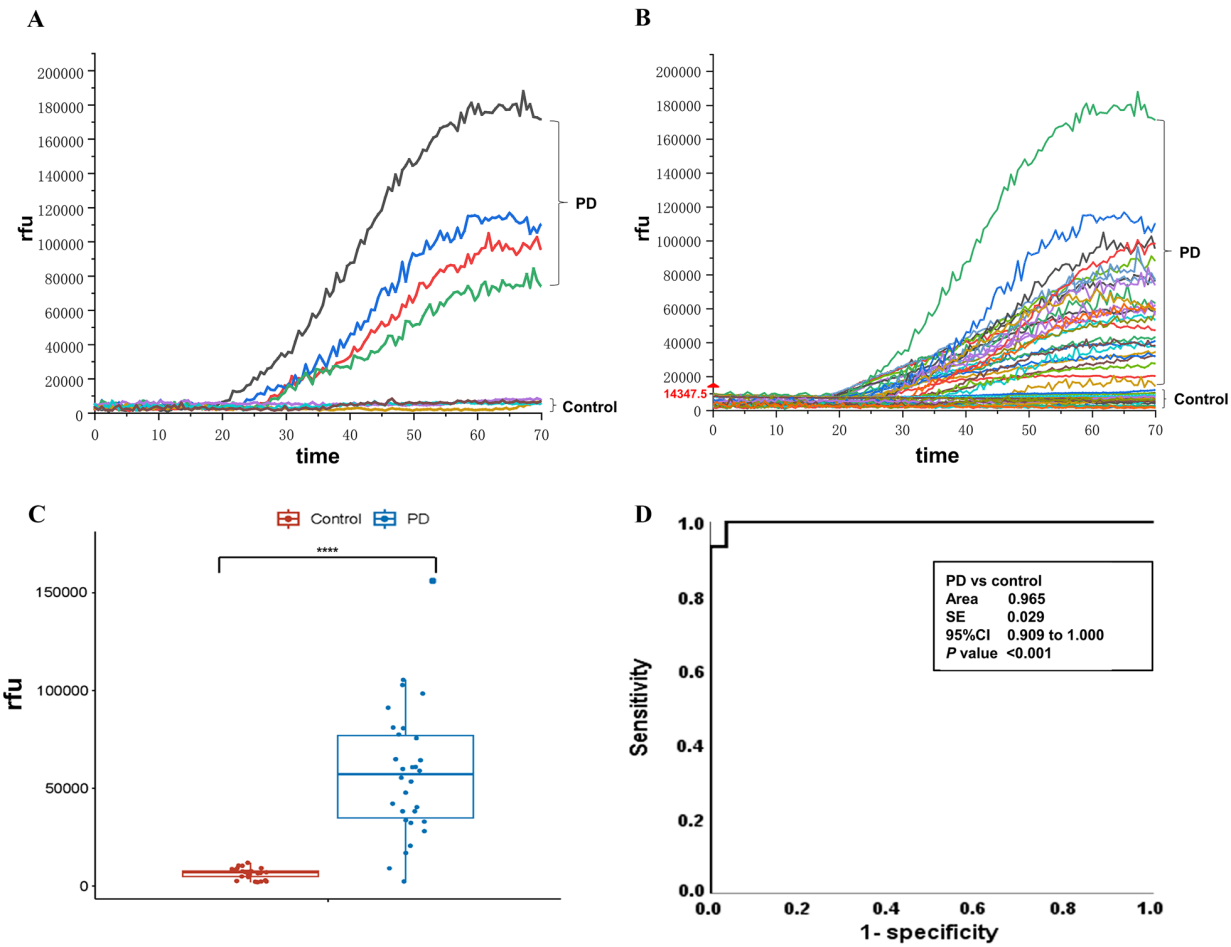
Detection of α-Syn seeding activity by skin RT-QuIC assay in patients with PD and controls. **A** RT-QuIC detection of α-Syn seeding activity in the cervical skin homogenates from 4 patients with PD and 4 controls. **B** RT-QuIC detection of α-Syn seeding activity in the skin homogenates of 30 patients and 28 controls. **C** Scatter graph of maximal thioflavin T (ThT) fluorescence at 70 h of the α-Syn seeding activity by RT-QuIC assay. **D** ROC curve and AUC calculation for comparison of α-Syn seeding activity between patients with PD and controls (*P* < 0.001)

First, we assessed the seeding activity of α-Syn in the samples from Cohort 1. The RT-QuIC assay successfully detected α-Syn seeding activity in the skin samples obtained from patients with PD. In contrast, no seeding activity was observed in the skin samples of the controls, even after a testing period of up to 70 h (Fig. 1A). To further assess the diagnostic value of our skin α-Syn RT-QuIC assay, we expanded our investigation to include biopsy samples obtained from PD patients (n = 30) and controls (*n* = 28) of Cohort 2. Remarkably, 28 out of 30 patients clinically diagnosed with PD displayed positive α-Syn seeding activity in their skin samples (sensitivity: 93.3%). Conversely, all control samples tested negative for seeding activity, yielding a specificity of 100.0% (Fig. 1B). Furthermore, we observed significantly higher maximum responses in the biopsy skin samples from PD patients compared to those from controls (*P* < 0.0001; Fig. 1C). Through the receiver operating characteristic (ROC) analysis, our skin-based assay demonstrated exceptional differentiating ability between PD patients and controls, with a notably high area under the ROC curve (AUC) value of 0.965. The calculation of the AUC was performed using a specific cut-off value of 14,347.5 (mean [SE] area under the curve [AUC]: 0.965 [0.029]; 95% confidence interval [CI] 0.909–1.000;* P* < 0.001; Fig. 1D).

The skin matrix in positive samples might hinder the spontaneous aggregation of α-Syn, leading to false negatives. We observed a notable reduction in seeding activity of 2 μl of a 10^–3^ α-Syn fibril (artificial seeds), when skin homogenates from the negative control group were added to artificial seeds, in comparison to conditions without skin homogenates (Additional file 1: Figure S2). This suggests the potential presence of a substance in the skin homogenate inhibiting α-Syn aggregation.

## Detection of fibrillary α-Syn in the RT-QuIC product of PD patients

### Transmission electron microscopy (TEM) and atomic force microscopy (AFM)

**Fig. 2:**
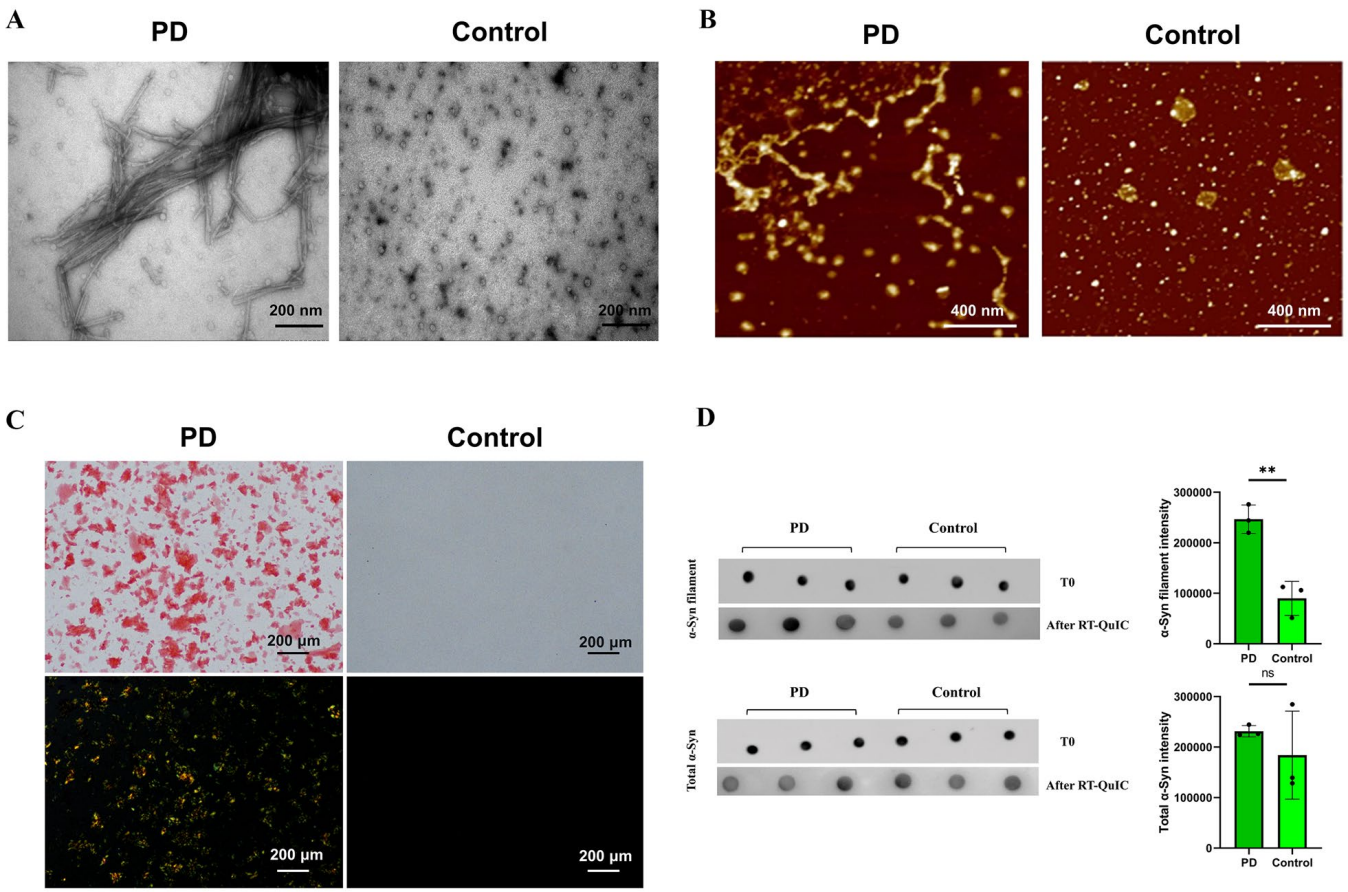
Detection of fibrillary α-Syn in the RT-QuIC product of PD patients. **A**, **B** The structural features of α-Syn aggregates extracted from PD-seeded RT-QuIC reactions were analyzed by employing transmission electron microscopy (TEM) and atomic force microscopy (AFM), which could not be observed in the control samples. **C** Congo red staining, observed under polarized light, exhibited an apple-green color in PD patients’ samples obtained from the RT-QuIC assay, whereas the control samples without PD showed a negative result. The scale bar represents 200 μm. **D** Analysis of Dot Blot both T0 and RT-QuIC Reaction in PD and Control Groups: Dot blot analysis was conducted on skin samples from three PD and three controls at both T0 and after RT-QuIC reactions using α-Syn filament conformation-specific antibodies (top panel) and total α-Syn antibodies (bottom panel). Densitometric quantification of α-Syn filament conformation-specific and total α-Syn levels revealed significantly increased levels of α-Syn filaments for the PD group compared to the control group, while total αSyn levels did not differ between groups. Results were analyzed using Student’s two-sample *t*-test. **p < 0.01 and not significant (ns) when *P* > 0.05

After conducting TEM and AFM analyses, we evaluated the structural characteristics of α-Syn fibrils amplified through skin RT-QuIC in multiple PD and control reaction product samples. The obtained images of fibril structures derived from PD patients are presented in Fig. 2A and B, revealing the presence of pathological α-Syn capable of seeding activity. In contrast, controls did not exhibit the formation of α-Syn fibrils in the seeding assay. Additional results for more samples are provided in the supplementary files (Additional file 1: Figure S3A, B).

### Congo red staining

To visualize the reaction products of the RT-QuIC assay, Congo red staining was applied to multiple PD and control reaction productions. The results demonstrated positive Congo red staining in the RT-QuIC productions of PD patients, exhibiting the characteristic apple-green birefringence under polarized light (Fig. 2C, Additional file 1: Figure S3C). This finding strongly indicates the generation of a substantial number of fibrils in PD patients during RT-QuIC detection.

### Dot Blot Using an α-syn filament conformation-specific antibody

To assess the presence of α-Syn filamentous conformations in the skin of PD patients compared to a control group, dot blot analysis was employed. Three skin samples exhibiting positive seeding activity for PD and three control group skin samples were arbitrarily selected for analysis both before (T0) and after the RT-QuIC reaction. A specific antibody targeting the filamentous structures of α-Syn was utilized for dot blot staining. Immunoblot analysis revealed PD patients displayed a notably heightened staining depth in comparison to the control group. Immunoblotting was also conducted using an antibody directed against total α-Syn, capable of detecting both native and misfolded forms. Immunoblot analysis demonstrated a similarity in total α-Syn levels between PD patients and the control group, consistent with previous reports (Fig. 2D).

### α-Syn seeding activity in skin biopsies correlates with disease duration and stage

**Fig. 3:**
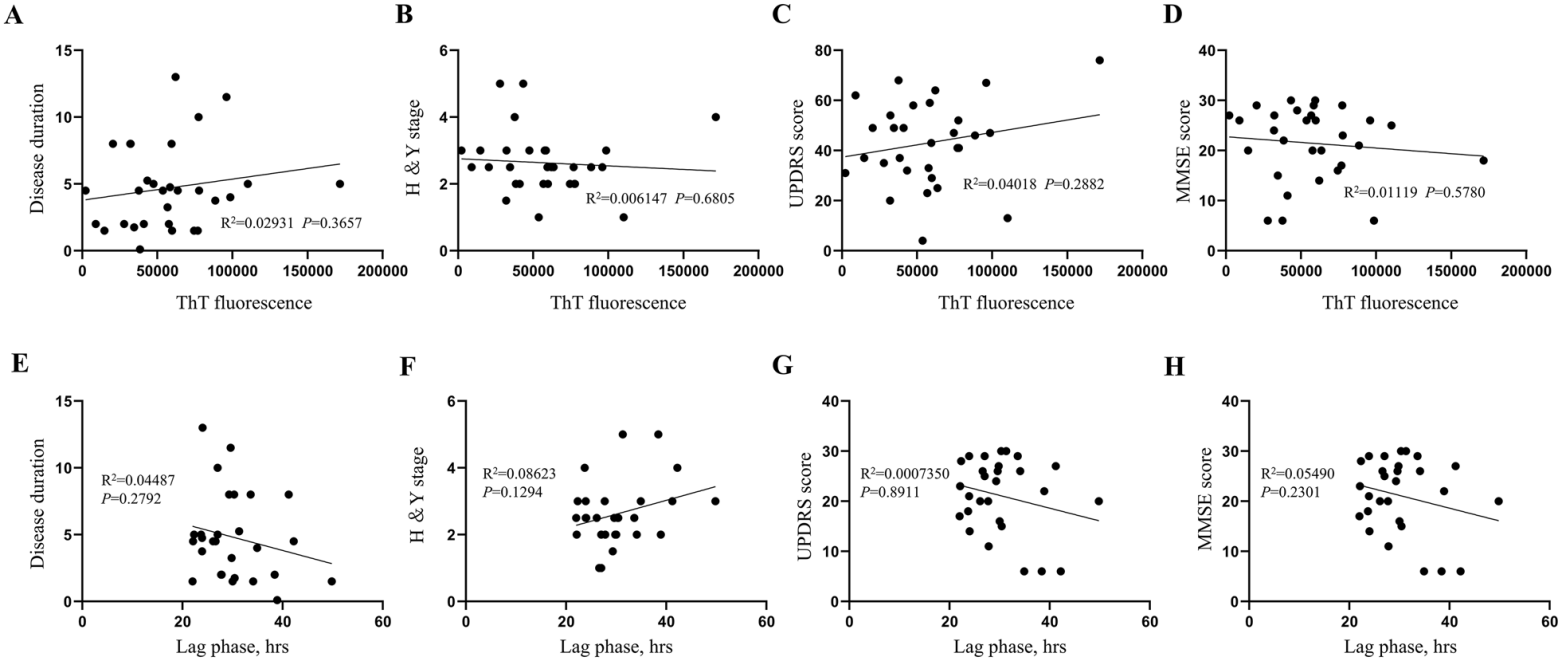
Skin RT-QuIC results exhibit no correlation with disease progression. Raw RT-QuIC assay parameters reveal no association with PD duration: longer disease duration (**A**, **E**), higher Hoehn and Yahr (H&Y) stage (**B**, **F**), higher Unified Parkinson’s Disease Rating Scale (UPDRS) score (**C**, **G**), and lower Mini-Mental State Examination (MMSE) score (**D**, **H**) do not exhibit a higher final ThT fluorescence or longer lag phase

The final fluorescence values of ThT and the lag phase (time to cross threshold fluorescence) in RT-QuIC were not found to be significantly correlated with the progression of the disease, Hoehn and Yahr (H&Y) stage, UPDRS score, and MMSE score (Fig. 3). Specifically, there were no significant correlations observed between disease duration (*r*^2^ = 0.02931, *p* = 0.3657), H&Y stage (*r*^2^ = 0.006147, *p* = 0.6805), UPDRS score (*r*^2^ = 0.04018, *p* = 0.2882), and MMSE score (*r*^2^ = 0.01119, *p* = 0.5780) with the final ThT fluorescence. Furthermore, disease duration (r^2^ = 0.04487,* p* = 0.2792), H&Y stage (*r*^2^ = 0.08623, *p* = 0.1294), UPDRS score (*r*^2^ = 0.0007350, *p* = 0.8911), and MMSE score (r^2^ = 0.05490, *p* = 0.2301) exhibited a lack of correlation with the lag phase.

## Discussion

The primary event in the pathogenesis of α-synucleinopathies, which include PD, DLB, and MSA, is the accumulation and deposition of misfolded α-Syn aggregates in the brain [1, 13]. However, the levels of pathological α-Syn in various bodily fluids often fall below the detection threshold of traditional biochemical methods. Misfolded α-Syn can induce misfolding of its native counterpart, both within cells and between cells, exhibiting prion-like seeding behavior [14]. RT-QuIC, an extremely sensitive protein amplification assay initially developed for prion disease detection, has shown promising results in detecting α-synucleinopathies [3]. Due to its similar embryonic origin to neural tissue and its close association with the CNS, the skin is considered a favorable site for investigating PD. Based on this, several research teams abroad have successfully applied skin seed amplification assays (SAA) to detect the seeding activity of α-Syn for the diagnosis of PD. We conducted a study to evaluate the clinical diagnostic applicability of skin α-Syn RT-QuIC assay in patients with PD from Chinese patients. Our findings demonstrate that skin α-Syn RT-QuIC detection can accurately diagnose PD in the Chinese population.

First, through repeated experimentation, we systematically determined the optimal concentration of SDS for the reaction. Subsequently, we performed preliminary detection using the RT-QuIC technique on small amounts of skin homogenate samples from clinically diagnosed PD patients and control subjects. These tests yielded a remarkable 100% sensitivity and specificity in detecting seeding activity. Then, we expanded our analysis to include 30 PD patients and 28 control samples. In this larger sample set, we achieved a specificity of 100% and a sensitivity of 93.3%. These findings support the potential of our skin α-Syn RT-QuIC assay as a valuable diagnostic tool for distinguishing PD patients from non-PD individuals. To further evaluate the RT-QuIC products, we employed several methods. Through the use of TEM and AFM, we were able to visualize the formation of rod-shaped fibrils in the skin production of PD patients. These observations, as depicted in the TEM and AFM images, provide compelling evidence that supports the pathological nature of α-Syn fibrils present in the skin of PD patients. The apple green polarization observed in PD patients’ samples, indicative of Congo red binding to amyloid fibrils, provides further confirmation of the pathological aggregation of α-Syn in PD. Additionally, when α-Syn fibrils were incubated with specific antibodies obtained from PD patient skin RT-QuIC productions, the resulting spot signals were significantly stronger compared to those of the control group. These findings indicate a considerably higher abundance of misfolded α-Syn in PD patients. However, the control group exhibited weak staining intensity for the α-Syn filament conformation. This disparity might be attributed to non-PD-related factors, such as sample processing, antibody specificity, and experimental conditions. Further research is necessary to investigate these potential influences thoroughly. Overall, these discoveries underscore the presence of pathological α-Syn conformations in the skin of PD patients, thereby providing further support for the involvement of misfolded α-Syn in the development of PD. These findings significantly contribute to our understanding of the molecular mechanisms involved in PD and highlight the potential of skin-based assays for studying α-Syn pathology.

One of the CSF α-Syn RT-QuIC analyses [4] revealed a correlation between α-Syn seeding activity and the H&Y stage, while others [15, 16] could not confirm it. Research from Kuzkina [17] demonstrated the skin α-Syn RT-QuIC seeding activity moderately correlated with the H&Y stage and duration of disease. Manne [6] conducted a correlation analysis to assess the relationship between RT-QuIC and various PD patient indicators. He found that the RT-QuIC values were significantly correlated with Unified LB stage *(P* < 0.0001), pathological diagnosis (*P* < 0.0001), MMSE scores (*P* = 0.0035), UPDRS scores (*P* < 0.0001), and disease duration (*P* = 0.0029). These results suggest an increase in α-Syn aggregation in peripheral tissues during disease progression, indicating the potential utility of skin RT-QuIC for monitoring disease advancement. Regrettably, our study did not identify a significant correlation between RT-QuIC seeding activity and disease duration, H&Y staging, UPDRS scores, or MMSE scores. Future prospective studies with biopsy assessments at multiple time points are warranted to further validate the correlation with disease stages and duration.

The deposition of cutaneous α-Syn in PD predominantly affects the autonomic nervous system [18, 19], and previous studies have implicated its association with dysautonomia [20]. Various investigations have consistently demonstrated the presence of α-Syn aggregates in multiple peripheral nervous system locations, including the skin, gastrointestinal tract, olfactory mucosa, and submandibular glands [6–8, 21, 22]. These findings suggest that the involvement of different parts of the peripheral nervous system may occur concurrently. Dysautonomia has been recognized as a crucial determinant of the ‘diffuse malignant’ phenotype in PD, characterized by a heightened symptom burden and accelerated disease progression [23]. The study by Kuzkina [17] revealed a significant correlation between higher α-Syn seeding activity of synaptic α-Syn in RT-QuIC and the presence of RBD, cognitive impairment, and constipation. The elevated skin α-Syn seeding activity, serving as an indicator of increased peripheral and potentially overall α-Syn aggregate burden, in patients exhibiting a “malignant phenotype,” suggests a potential utility for skin RT-QuIC assay in identifying PD subgroups with distinct rates of disease progression.

No detectable seeding activity of α-Syn was found in the skin samples from two clinically diagnosed PD patients. Several factors may contribute to these negative results, including the sample collection, the sensitivity of the technique, experimental conditions, procedural techniques, sample quality, and interference from substances within the skin. The findings from the skin study could potentially imply that the disease may have distinct presentations or subtypes, some of which might not be effectively captured through skin-based assays alone. This could suggest that relying solely on skin samples for diagnostic or prognostic purposes might lead to incomplete conclusions in certain cases. In addition, the pathological distribution of α-Syn in the skin is not uniform, and skin biopsies often collect relatively small tissue samples. If the “seeds” are not obtained, it may lead to negative results. An alternative scenario is that RT-QuIC-negative PD patients may manifest a proposed central nervous system-predominant phenotype, wherein basal ganglia pathology is hypothesized to precede involvement of the peripheral nervous system. Consequently, these patients may not show signs of peripheral nervous system damage. Given the limited number of cases in our study, further research is needed to investigate the reasons for obtaining negative results in skin RT-QuIC assay for clinical diagnosis of PD patients. Additionally, for the two PD patients, the relative fluorescence unit (RFU) values are higher than but very close to the threshold values. This suggests a possibly lower concentration of "seeds" in the skin homogenate, resulting in lower ThT fluorescence values. Upon reviewing the clinical data of these two patients, it was discovered that they were not in the early stage of PD, indicating a potential lack of significant correlation between a load of skin “seeds” and clinical disease progression. Further research is necessary to analyze the relationship between α-Syn seeding activity and the progression and severity of PD in patients.

Several studies have reported abnormal accumulation of α-Syn in peripheral tissues and biological fluid, such as blood [24], olfactory mucosa [21, 25], and urine [26] of patients with α-synucleinopathies. The latest research findings indicate that the serum from α-synucleinopathies exhibits a seeding ability. Moreover, the detection of α-Syn “seeds” amplified from serum through RT-QuIC emerges as a highly effective biomarker for diagnosing α-synucleinopathies [24]. This discovery has opened up possibilities for non-invasive sample collection from patients with α-synucleinopathies, utilizing RT-QuIC amplification technology. By analyzing easily accessible peripheral tissues without invasive procedures, the diagnosis of α-synucleinopathies can be achieved. Furthermore, obtaining specimens at different disease stages enables monitoring of α-Syn’s seeding activity, as well as its biochemical and structural characteristics in response to specific drugs. This non-invasive approach shows promise for enhancing the diagnosis and treatment of neurodegenerative diseases in the future.

Our study possesses certain limitations. Firstly, the inclusion of a relatively small number of PD and control cases is noteworthy. To enhance the robustness of our findings, future investigations should incorporate larger cohorts for validation purposes. Furthermore, an additional limitation of our study pertains to the assessment of sensitivity and specificity of α-Syn seeding activity relied on the clinical diagnosis of PD and non-PD, not by brain biopsy or autopsy results.

## Conclusion

In conclusion, we have effectively applied RT-QuIC technology to detect misfolded α-Syn seeding activity in skin samples obtained from individuals in the Chinese population diagnosed with PD. Our study showcases high specificity and sensitivity, establishing a robust basis for its prospective utilization as a biomarker for PD.

### Supplementary Information

**Additional file 1**: **Figure S1.** The Impact of SDS Concentrations on RT-QuIC Reaction. Positive control: 2 μl of a 1:1,000 dilution of α-Syn fibrils. Various SDS concentrations (0.00125%, 0.001%, 0.00075%, 0.0005%, 0%) were added. At 0.00125% SDS, positive control fluorescence increased over 10 hours, reaching a peak of ~130,000. At 0.001%, 0.00075%, 0.0005%, and 0% SDS, fluorescence intensity decreased. The negative control showed increased fluorescence only at 0.00125% SDS. **Figure S2.** RT-QuIC Reaction Curves for Diluted α-Syn Fibrils and Fibrils with Negative Control Skin Matrix. Negative control groups plateau at 40,000 ThT fluorescence around 20 h. Diluted α-Syn fibrils show a sharp increase starting at 10 h, reaching a peak ThT fluorescence of 160,000. **Figure S3**. Biochemical and Morphological Analyses of RT-QuIC Products. (Figure S3A, B): AFM (The scale bar represents 400 nm) and TEM (The scale bar represents 200 nm) images reveal fibrillary structures in PD-seeded reactions, while control samples show no fibril formation. (Figure S3C): Congo red staining highlights amyloid aggregates in PD-seeded reactions, with control samples lacking observable amyloid structures. The scale bar represents 200 μm. **Figure S4.** ROC Curve Analysis of α-Syn Seeding Activity. ROC curve analysis comparing α-Syn seeding activity between PD and controls reveals a significant difference in normalized data (*P*<0.001). **Figure S5.** Representative Patients' Reaction Wells with Relative Fluorescence Units (RFU).

## Data Availability

All data generated by the current study are available upon reasonable request.
