# Supplementary material for: Detection of skin α-synuclein using RT-QuIC as a diagnostic biomarker for Parkinson’s disease in the Chinese population
Source: Eur J Med Res. 2024 Feb 9;29:114. doi: 10.1186/s40001-024-01705-x (PMC10854029; doi:10.1186/s40001-024-01705-x)
Supplement: Supplementary file 1 — Additional file 1: Figure S1. The Impact of SDS Concentrations on RT-QuIC Reaction. Positive control: 2 μl of a 1:1,000 dilution of α-Syn fibrils. Various SDS concentrations (0.00125%, 0.001%, 0.00075%, 0.0005%, 0%) were added. At 0.00125% SDS, positive control fluorescence increased over 10 hours, reaching a peak of ~130,000. At 0.001%, 0.00075%, 0.0005%, and 0% SDS, fluorescence intensity decreased. The negative control showed increased fluorescence only at 0.00125% SDS. Figure S2. RT-QuIC Reaction Curves for Diluted α-Syn Fibrils and Fibrils with Negative Control Skin Matrix. Negative control groups plateau at 40,000 ThT fluorescence around 20 h. Diluted α-Syn fibrils show a sharp increase starting at 10 h, reaching a peak ThT fluorescence of 160,000. Figure S3. Biochemical and Morphological Analyses of RT-QuIC Products. (Figure S3A, B): AFM (The scale bar represents 400 nm) and TEM (The scale bar represents 200 nm) images reveal fibrillary structures in PD-seeded reactions, while control samples show no fibril formation. (Figure S3C): Congo red staining highlights amyloid aggregates in PD-seeded reactions, with control samples lacking observable amyloid structures. The scale bar represents 200 μm. Figure S4. ROC Curve Analysis of α-Syn Seeding Activity. ROC curve analysis comparing α-Syn seeding activity between PD and controls reveals a significant difference in normalized data (P<0.001). Figure S5. Representative Patients' Reaction Wells with Relative Fluorescence Units (RFU). [file 40001_2024_1705_MOESM1_ESM.zip › New folder/Supplementary files.docx]

**Figure legend:**

**Figure 1:**  The Impact of SDS Concentrations on RT-QuIC Reaction: Positive control: 2 μl of a 1:1,000 dilution of α-Syn fibrils. Various SDS concentrations (0.00125%, 0.001%, 0.00075%, 0.0005%, 0%) were added. At 0.00125% SDS, positive control fluorescence increased over 10 hours, reaching a peak of ~130,000. At 0.001%, 0.00075%, 0.0005%, and 0% SDS, fluorescence intensity decreased. The negative control showed increased fluorescence only at 0.00125% SDS.

**Figure 2:** RT-QuIC reaction curves for diluted α-Syn fibrils and fibrils with negative control skin matrix. Negative control groups plateau at 40,000 ThT fluorescence around 20 hours. Diluted α-Syn fibrils show a sharp increase starting at 10 hours, reaching a peak ThT fluorescence of 160,000.

**Figure 3:** Biochemical and morphological analyses of RT-QuIC products were conducted using AFM, TEM, and Congo red staining. (Figure 3A-B): AFM (The scale bar represents 400 nm) and TEM (The scale bar represents 200 nm) images reveal fibrillary structures in PD-seeded reactions, while control samples show no fibril formation. (Figure 3C): Congo red staining highlights amyloid aggregates in PD-seeded reactions, with control samples lacking observable amyloid structures. The scale bar represents 200 μm.

**Figure 4:** ROC curve analysis comparing α-Syn seeding activity between PD and controls reveals a significant difference in normalized data (*P*<0.001).

**Figure 5:** Illustration of representative patients' individual reaction wells with relative fluorescence units (RFU).
